# A feasibility study to identify proteins in the residual Pap test fluid of women with normal cytology by mass spectrometry-based proteomics

**DOI:** 10.1186/1559-0275-11-30

**Published:** 2014-07-14

**Authors:** Kristin LM Boylan, Somaieh Afiuni-Zadeh, Melissa A Geller, Kayla Hickey, Timothy J Griffin, Stefan E Pambuccian, Amy PN Skubitz

**Affiliations:** 1 Department of Laboratory Medicine and Pathology, University of Minnesota, MMC 395, 420 Delaware St. S.E., Minneapolis, MN 55455, USA; 2 Department of Obstetrics, Gynecology, and Women’s Health, Division of Gynecologic Oncology, University of Minnesota, Minneapolis, MN, USA; 3 Department of Biochemistry, Molecular Biology and Biophysics, University of Minnesota, Minneapolis, MN, USA; 4 Department of Pathology, Loyola University Medical Center, Chicago, IL, USA

**Keywords:** Mass spectrometry, Proteomics, Pap test, Biomarker discovery

## Abstract

**Background:**

The proteomic analysis of body fluids is a growing technology for the identification of protein biomarkers of disease. Given that Papanicolaou tests (Pap tests) are routinely performed on over 30 million women annually in the U.S. to screen for cervical cancer, we examined the residual Pap test fluid as a source of protein for analysis by mass spectrometry (MS). In the liquid-based Pap test, cervical cells are collected from the ectocervix and placed into an alcohol-based fixative prior to staining and pathologic examination. We hypothesized that proteins shed by cells of the female genital tract can be detected in the Pap test fixative by MS-based proteomic techniques. We examined the feasibility of using residual fluid from discarded Pap tests with cytologically “normal” results to optimize sample preparation for MS analysis. The protein composition of the cell-free Pap test fluid was determined by silver staining of sodium dodecyl sulfate -polyacrylamide gels, and the abundance of serum proteins was examined by Western immunoblot using an antibody against human serum albumin. Both pooled and individual samples were trypsin digested and analyzed by two-dimensional MS/MS. Proteins were identified by searching against the Human Uniprot database, and characterized for localization, function and relative abundance.

**Results:**

The average volume of the residual Pap test fluid was 1.5 ml and the average protein concentration was 0.14 mg/ml. By Western immunoblot we showed that the amount of albumin in each sample was significantly reduced compared to normal serum. By MS/MS, we identified 714 unique proteins in pooled Pap test samples and an average of 431 proteins in individual samples. About 40% of the proteins identified were extracellular or localized to the plasma membrane. Almost 20% of the proteins identified were involved in immunity and defense, characteristic of the healthy cervical-vaginal proteome. By merging the protein sets from the individual and pooled Pap test samples, we created a “Normal Pap test Core Proteome” consisting of 153 proteins.

**Conclusions:**

Residual Pap test fluid contains a sufficient amount of protein for analysis by MS and represents a valuable biospecimen source for the identification of protein biomarkers for gynecological diseases.

## Background

Screening for cervical cancer by Papanicolaou tests (Pap tests) has been routinely performed for over 50 years [1]. The liquid-based Pap test consists of collecting cervical cells from the ectocervix and placing them into a vial containing a fluid transport medium to preserve the cells [2,3]. Two FDA approved liquid-based Pap tests are widely used for the screening and detection of cervical cancer, pre-cancerous lesions, and atypical cells [4]. One Pap test, which we used in this study, is the SurePath^TM^ Pap test [Becton-Dickinson (BD Diagnostics, Burlington, NC)] which has an alcohol-based fixative consisting of 21.7% ethanol, 1.2% methanol, 1.1% isopropanol, and formaldehyde [5]. The second Pap test, the ThinPrep Pap test (Hologic, Inc., Bedford, MA) contains 30-60% methanol as the fixative [6]. In each case, fixative is removed from the vials and undergoes automated processing so that the cells are stained on a slide, and then examined by a pathologist to identify the presence of premalignant and malignant cells. The liquid fixative solution in which the cells are collected for Pap tests is routinely discarded after examination of the cells. Over 30 million Pap tests are analyzed annually by cytopathologists [4,7-9]; making this an abundant source of samples for experimentation and for the potential detection of a variety of gynecological diseases in the future. To our knowledge, no one has analyzed the residual Pap test fluid by the latest mass spectrometry (MS)-based proteomic techniques to identify proteins or potential biomarkers of disease.

Several groups have performed mass spectrometry-based proteomic analysis of cervical-vaginal fluid obtained using swabs, gauze, or Dacron-tipped plastic applicators (reviewed in [10]). Cervical-vaginal fluid is a complex biological fluid that protects and lubricates the endometrial, cervical and vaginal lining. This fluid contains proteins predominantly synthesized by the endocervix and vaginal cells, but it has been shown to also contain proteins from amniotic fluid leakage during pregnancy, from endometrial and tubal secretions, and the peritoneal fluid [11-15]. Studies have attempted to define the proteome of healthy women as well as identify potential markers for preterm birth, pregnancy, and intra-amnionic infection [10,11,13-21]. However, to date, the use of residual Pap test fluid as a source for proteomics and biomarker discovery has not been reported.

The primary objective of this study was to determine whether residual Pap test fixative is a suitable source of protein for mass spectrometry-based proteomic techniques. We have quantified the concentration of protein present in the residual SurePath^TM^ fixative of Pap tests taken from over 100 women with normal cytology. We developed a protocol for processing the residual Pap test fluid so that peptides can be analyzed by MS/MS and proteins identified from the Human Uniprot database. Finally, we found extensive overlap between the proteins that we define as our “Normal Pap test Core Proteome” and lists of cervical-vaginal fluid proteins identified by others using different sampling methods [10,11,13-15,17-22].

## Results

### Cell-free residual Pap test fluid contains protein

**Figure 1:**
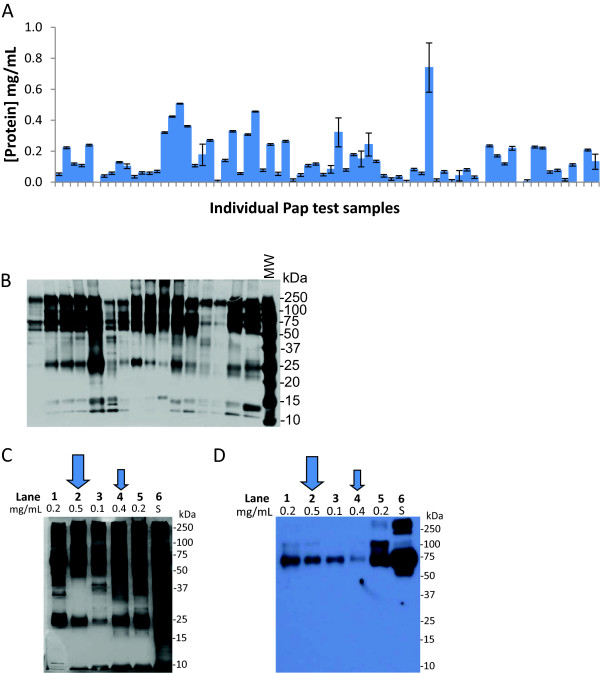
**Protein content of residual Pap test fluid. (A)** The protein concentration of residual Pap test fluid from 72 randomly selected SurePath^TM^ samples was measured using the BCA protein assay in duplicate. Error bars show standard deviation. **(B)** The protein composition of the cell-free Pap test fluid from 16 individuals randomly selected from the samples in **(A)** was visualized by silver staining of SDS-PAGE gels. Five micrograms of protein was loaded per lane. MW, molecular weight standards. **(C)** Five micrograms of protein from serum (lane 6; S) or 5 different individuals’ SurePath^TM^ samples (lanes 1–5) with varying protein concentrations (0.1–0.5 mg/ml) were visualized by silver stained SDS-PAGE. **(D)** In a parallel experiment, the SDS-PAGE gels was transferred to a PVDF membrane and probed by Western immunoblot with a polyclonal antibody recognizing human serum albumin. In **(C)** and **(D)**, the protein concentration of the individual’s Pap test sample is listed above each lane. The large arrow indicates the individual’s Pap test sample with the highest protein concentration (lane 2; 0.5 mg/ml), and the small arrow indicates the individual’s Pap test sample with the lowest serum albumin content as detected by Western immunoblotting (lane 4).

To determine whether the cell-free fluid remaining after the examination of cervical cells from the SurePath^TM^ liquid based Pap test preparation contained sufficient protein for mass spectrometry analysis, we measured the volume and protein content of over 100 residual SurePath^TM^ samples. On average, these samples contained 1.5 ml of SurePath^TM^ fixative. The protein concentration in 72 of the samples was determined using the bicinchoninic acid (BCA) protein assay (Pierce Protein Research Products, Rockford, IL) on duplicate samples and ranged from undetectable to more than 0.7 mg/ml; with an average protein concentration of 0.14 mg/ml (Figure 1A). Sixteen of these 72 Pap test fixative samples were randomly selected to be examined by sodium dodecyl sulfate (SDS)-polyacrylamide gel electrophoresis (PAGE). We found many protein bands visible by silver stain, indicating the presence of both high and low abundance proteins in residual Pap test fluid (Figure 1B). Overall, the protein patterns appeared relatively similar in number, size, and intensity among the individual samples. Several major protein bands of 50–250 kD were detected in almost all of the samples, as well as proteins of ~25 kD and 10–15 kD.To determine whether the variation in protein concentration of the residual Pap test fluid was due to contamination of the samples with blood proteins, we separated the proteins from the residual Pap test fluid of five individuals by size on SDS-PAGE (Figure 1C) and then performed Western immunoblot analysis of the protein using an antibody to human serum albumin (Figure 1D). Comparison of an equal amount of serum (lane 6; S) to the protein in the residual Pap test fluid (lanes 1–5) showed the variable presence of albumin in each of the residual Pap test samples, however at a substantially lower level than was found in serum (lane 6). The results of the Western immunoblot analysis also demonstrated that the protein concentration of the residual Pap test fluid did not directly correlate with the level of serum albumin present. For example, the sample with the highest protein concentration of 0.5 mg/ml (Figure 1C and D, lane 2, large arrow) did not contain more serum albumin than the other samples. Similarly, the sample in which the least amount of serum albumin was detected (Figure 1C and D, lane 4, small arrow) had the second highest protein concentration of 0.4 mg/ml.

### Mass spectrometry of pooled Pap test samples

**Figure 2:**
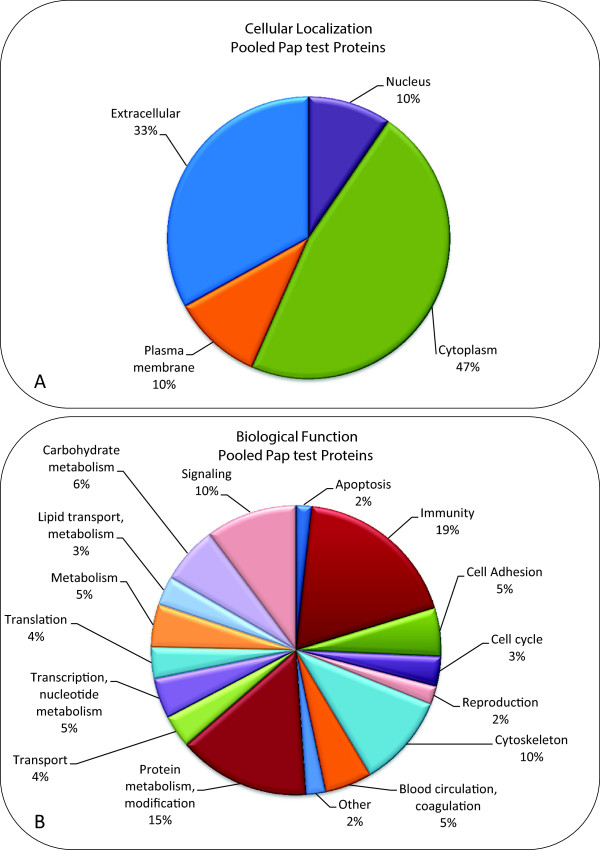
**Classification of proteins in the pooled Pap test samples by cellular localization and function.** The proteins in the two pooled samples were combined into one group of 714 proteins. These proteins were then classified by cellular localization and biological function using PANTHER database (version 8.1) and Ingenuity IPA (version 2013, 17199142) and the UniProtKB Protein Knowledge database. **(A)** Most of the proteins (608/714) were classified according to cellular localization. The remaining 106 proteins were unclassified. **(B)** Most of the proteins (685/714) were classified according to biological function. The remaining 29 proteins were unclassified.

In order to get an overview of the proteins present in the SurePath^TM^ fluid, we pooled residual Pap test fluid from 40 women with normal cervical cytology for analysis by 2D tandem mass spectrometry. These 40 samples were selected from the 56 samples that remained from the original 72 samples (Figure 1A), after 16 samples were used for SDS-PAGE analysis (Figure 1B). The selection of these 40 samples was based solely on the fact that they contained >50 ug of protein. A total of 714 unique proteins were identified when the pooled samples were run in two separate experiments (see Additional file 1). Only proteins from UniProtKB/Swiss-Prot (reviewed) are reported in Additional file 1. The cellular localization of the 714 proteins was determined using Gene Ontology (GO) classifications (Figure 2A) [23]. Over 40% of the proteins identified in the pooled Pap test samples were extracellular proteins or plasma membrane proteins. The remaining proteins were cytoplasmic or nuclear proteins, suggesting the occurrence of cell lysis *in situ*. The proteins identified in the pooled Pap test samples were also classified according to several general functional terms by the PANTHER classification system (Figure 2B) [24] and grouped into over a dozen categories. The major functional groups contained proteins involved in immunity and defense (19%), protein metabolism and modification (15%), the cytoskeleton (10%), and other cellular processes such as cell signaling (10%) and cell adhesion (5%). Minor groups of proteins were involved in transport (4%), cell cycle (3%), and reproduction (2%).

### Sources of variability in mass spectrometry analysis

In LC/MS proteomic studies, several sources of variability exist, including biological, technical, and experimental [25,26]. In order to address the issue of technical variability which occurs during sample preparation (including trypsin digestion and solid phase extraction clean up), we randomly selected a Pap test sample from a healthy individual and precipitated the protein with acetone. The protein was then divided into two identical aliquots. These two samples were then digested by the filter aided sample preparation FASP technique in parallel and then these replicates were analyzed by LC/MS. The average of all standard deviations calculated for each protein in the replicates was calculated to have a variance of 1.23×10^-3^ with a CV of 19.23%. We then performed independent injections of one aliquot in three different MS runs; the short-term run-to-run instrumental variance was estimated to be 5.69×10^-4^. These results are comparable to values obtained in the literature [25,26].

### Mass spectrometry of individual Pap test samples

**Figure 3:**
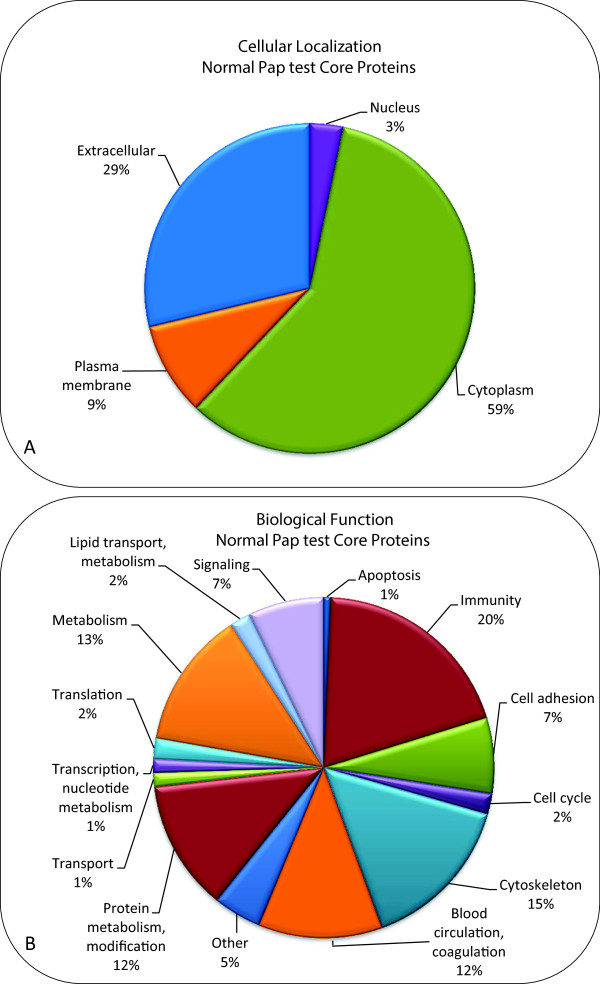
**Classification of proteins in the “Normal Pap test Core Proteome” by cellular localization and function.** The 153 proteins that comprised the “Normal Pap test Core Proteome”, as defined by their presence in 4 of the 5 individual’s Pap tests, were classified by cellular localization and biological function using PANTHER database (version 8.1), Ingenuity IPA (version 2013, 17199142), and the UniProtKB Protein Knowledge database. **(A)** The proteins were classified according to cellular localization. **(B)** The proteins were classified according to biological function.

Five residual Pap test samples were randomly selected from a second cohort of 20 individuals with normal cytology and were prepared for mass spectrometry using the FASP technique (see Methods). On average, 431 proteins were identified in the individual samples (ranging from 317 to 539 proteins) (Table 1 and Additional file 2). Only proteins from UniProtKB/Swiss-Prot (reviewed) are reported in Additional file 2. Approximately 70% (60-85%) of the proteins identified in the individual samples were also found in the pooled samples (Table 1). The lists of proteins that were identified in the Pap test fluid from each of five individuals (Additional file 2) were then analyzed for their frequency of occurrence. The 153 proteins that were present in 4 of the 5 individuals are hereafter designated the, “Normal Pap test Core Proteome” and are listed in Table 2 with their Protein name, Gene name, and Swiss-Prot accession number. Classification of all 153 proteins in the “Normal Pap test Core Proteome” based on cellular localization (Figure 3A) shows that most of the proteins were derived from the cytoplasm (59%), and over one third of the proteins were extracellular (29%) or in the plasma membrane (9%), which is in agreement with the pooled sample cellular localization categories (Figure 2A). Functional classification of the 153 proteins in the “Normal Pap test Core Proteome” (Figure 3B) is also similar to the pooled samples and shows a great diversity of biological roles, in which immunity and defense (20%), cytoskeletal proteins (15%), and protein metabolism and modification (12%) are the largest categories (Figure 2B). One difference between the functional categories of proteins present in the Pooled Pap test and the “Normal Pap test Core Proteome” is the percentage of proteins involved in blood circulation and coagulation that were identified. In the “Normal Pap test Core Proteome”, 18/153 (12%) were categorized as functioning in blood circulation and coagulation. In contrast, in the Pooled Pap test samples, only 5% (36 of 685) of the proteins were in this category.

**Table 1:** Proteins identified in individual residual Pap test samples

	**Individual Pap test samples**				
	**NP94**	**NP134**	**NP137**	**NP929**	**NP933**
**Total number of proteins identified by MS/MS in this individual’s Pap test**^**(a)**^	321	317	539	500	479
**Number of this individual’s proteins also identified in the pooled samples**^**(b)**^	280	257	293	311	286
**Number of this individual’s proteins also identified in the “Normal Pap test Core Proteome” **^**(c)**^	131	124	137	152	152

Proteins were identified from the MS/MS data for each of the 5 individual Pap test samples from women with normal cytology.

^(a)^The total number of proteins identified in each individual Pap test was counted and the proteins listed in Additional file 2. False positive rates were < 1.0% for all experiments.

^(b)^The lists of proteins that were identified for each individual were compared to the list of 714 proteins identified in the pooled Pap test samples (listed in Additional file 1).

^(c)^The lists of proteins that were identified for each individual were compared to the list of 153 proteins identified in our newly defined “Normal Pap test Core Proteome” (listed in Table 2).

**Table 2:** “Normal Pap test Core Proteome,” defined as the 153 proteins that were identified by MS/MS in the residual Pap test fluid from 4 out of 5 women with normal cytology

	**Identified proteins**	**Gene name**	**Accession number**	Present in CVF Core Proteome*	Present in at least one other CVF Proteome study**
1	14-3-3 protein epsilon	YWHAE	[Swiss-Prot: P62258]		X
2	14-3-3 protein zeta/delta	YWHAZ	[Swiss-Prot: P63104]	*√*	
3	40S ribosomal protein S16	RPS16	[Swiss-Prot: P62249]		X
4	78 kDa glucose-regulated protein	HSPA5	[Swiss-Prot: P11021]	*√*	
5	Acid ceramidase	ASAH1	[Swiss-Prot: Q13510]		
6	Actin, cytoplasmic 1	ACTB	[Swiss-Prot: P60709]	*√*	
7	Acylamino-acid-releasing enzyme	APEH	[Swiss-Prot: P13798]		
8	Adenylate kinase 2, mitochondrial	AK2	[Swiss-Prot: P54819]		
9	Afamin	AFM	[Swiss-Prot: P43652]		X
10	Alpha-1-antitrypsin	SERPINA1	[Swiss-Prot: P01009]	*√*	
11	Alpha-1B-glycoprotein	A1BG	[Swiss-Prot: P04217]		X
12	Alpha-2-HS-glycoprotein	AHSG	[Swiss-Prot: P02765]	*√*	
13	Alpha-2-macroglobulin	A2M	[Swiss-Prot: P01023]		X
14	Alpha-actinin-1	ACTN1	[Swiss-Prot: P12814]		X
15	Alpha-actinin-4	ACTN4	[Swiss-Prot: O43707]	*√*	
16	Alpha-enolase	ENO1	[Swiss-Prot: P06733]	*√*	
17	Annexin A1	ANXA1	[Swiss-Prot: P04083]	*√*	
18	Annexin A11	ANXA11	[Swiss-Prot: P50995]		X
19	Annexin A2	ANXA2	[Swiss-Prot: P07355]	*√*	
20	Annexin A3	ANXA3	[Swiss-Prot: P12429]	*√*	
21	Annexin A5	ANXA5	[Swiss-Prot: P08758]		X
22	Apolipoprotein D	APOD	[Swiss-Prot: P05090]		X
23	Aspartate aminotransferase, cytoplasmic	GOT1	[Swiss-Prot: P17174]		X
24	Aspartate aminotransferase, mitochondrial	GOT2	[Swiss-Prot: P00505]		
25	Beta-2-glycoprotein 1	APOH	[Swiss-Prot: P02749]	*√*	
26	Brain acid soluble protein 1	BASP1	[Swiss-Prot: P80723]		X
27	Cadherin-1	CDH1	[Swiss-Prot: P12830]		X
28	Calmodulin-like protein 3	CALML3	[Swiss-Prot: P27482]	*√*	
29	Calpain-1 catalytic subunit	CAPN1	[Swiss-Prot: P07384]		X
30	Catalase	CAT	[Swiss-Prot: P04040]	*√*	
31	Cathepsin B	CTSB	[Swiss-Prot: P07858]	*√*	
32	Cathepsin D	CTSD	[Swiss-Prot: P07339]		X
33	Cathepsin G	CTSG	[Swiss-Prot: P08311]	*√*	
34	CD44 antigen	CD44	[Swiss-Prot: P16070]		
35	Ceruloplasmin	CP	[Swiss-Prot: P00450]	*√*	
36	Chitotriosidase-1	CHIT1	[Swiss-Prot: Q13231]		X
37	Complement C3	C3	[Swiss-Prot: P01024]	*√*	
38	Complement C4-A	C4A	[Swiss-Prot: P0C0L4]		X
39	Complement component C8 gamma chain	C8G	[Swiss-Prot: P07360]		
40	Complement decay-accelerating factor	CD55	[Swiss-Prot: P08174]		X
41	Complement factor H	CFH	[Swiss-Prot: P08603]	*√*	
42	Cystatin-B	CSTB	[Swiss-Prot: P04080]	*√*	
43	Cytochrome c	CYCS	[Swiss-Prot: P99999]		X
44	Dipeptidyl peptidase 4	DPP4	[Swiss-Prot: P27487]		X
45	Elongation factor 1-alpha 1	EEF1A1	[Swiss-Prot: P68104]	*√*	
46	Elongation factor 1-gamma	EEF1G	[Swiss-Prot: P26641]		X
47	Epididymal secretory protein E1	NPC2	[Swiss-Prot: P61916]		X
48	Erythrocyte band 7 integral membrane protein	STOM	[Swiss-Prot: P27105]		X
49	Ezrin	EZR	[Swiss-Prot: P15311]		X
50	Ferritin heavy chain	FTH1	[Swiss-Prot: P02794]		
51	Ferritin light chain	FTL	[Swiss-Prot: P02792]		X
52	Fibrinogen beta chain	FGB	[Swiss-Prot: P02675]	*√*	
53	Fibrinogen gamma chain	FGG	[Swiss-Prot: P02679]	*√*	
54	Fibronectin	FN1	[Swiss-Prot: P02751]		X
55	Fructose-bisphosphate aldolase A	ALDOA	[Swiss-Prot: P04075]	*√*	
56	Galectin-3-binding protein	LGALS3BP	[Swiss-Prot: Q08380]		X
57	Gamma-glutamylcyclotransferase	GGCT	[Swiss-Prot: O75223]	*√*	
58	Gelsolin	GSN	[Swiss-Prot: P06396]		X
59	Glutamine synthetase	GLUL	[Swiss-Prot: P15104]		X
60	Glutathione reductase, mitochondrial	GSR	[Swiss-Prot: P00390]		X
61	Glutathione synthetase	GSS	[Swiss-Prot: P48637]		
62	Glyceraldehyde-3-phosphate dehydrogenase	GAPDH	[Swiss-Prot: P04406]	*√*	
63	Haptoglobin	HP	[Swiss-Prot: P00738]	*√*	
64	Heat shock cognate 71 kDa protein	HSPA8	[Swiss-Prot: P11142]	*√*	
65	Heat shock protein beta-1	HSPB1	[Swiss-Prot: P04792]	*√*	
66	Heme-binding protein 2	HEBP2	[Swiss-Prot: Q9Y5Z4]		
67	Hemoglobin subunit alpha	HBA1	[Swiss-Prot: P69905]	*√*	
68	Hemoglobin subunit beta	HBB	[Swiss-Prot: P68871]	*√*	
69	Hemopexin	HPX	[Swiss-Prot: P02790]	*√*	
70	Histidine-rich glycoprotein	HRG	[Swiss-Prot: P04196]		X
71	Histone H4	HIST1H4A	[Swiss-Prot: P62805]	*√*	
72	Ig alpha-1 chain C region	IGHA1	[Swiss-Prot: P01876]	*√*	
73	Ig gamma-1 chain C region	IGHG1	[Swiss-Prot: P01857]	*√*	
74	Ig gamma-3 chain C region	IGHG3	[Swiss-Prot: P01860]		X
75	Ig lambda-2 chain C regions	IGLC2	[Swiss-Prot: P0CG05]		
76	Ig lambda-7 chain C region	IGLC7	[Swiss-Prot: A0M8Q6]		
77	Ig mu chain C region	IGHM	[Swiss-Prot: P01871]	*√*	
78	IgGFc-binding protein	FCGBP	[Swiss-Prot: Q9Y6R7]		
79	Immunoglobulin J chain	IGJ	[Swiss-Prot: P01591]	*√*	
80	Involucrin	IVL	[Swiss-Prot: P07476]	*√*	
81	Keratin, type I cytoskeletal 10	KRT10	[Swiss-Prot: P13645]		
82	Keratin, type I cytoskeletal 13	KRT13	[Swiss-Prot: P13646]		
83	Keratin, type I cytoskeletal 14	KRT14	[Swiss-Prot: P02533]		
84	Keratin, type I cytoskeletal 19	KRT19	[Swiss-Prot: P08727]		
85	Keratin, type II cytoskeletal 1	KRT1	[Swiss-Prot: P04264]		
86	Keratin, type II cytoskeletal 2	KRT2	[Swiss-Prot : P35908]		
87	Keratin, type II cytoskeletal 4	KRT4	[Swiss-Prot: P19013]		
88	Keratin, type II cytoskeletal 5	KRT5	[Swiss-Prot: P13647]		
89	Keratin, type II cytoskeletal 6A	KRT6A	[Swiss-Prot: P02538]		
90	Kininogen-1	KNG1	[Swiss-Prot: P01042]		X
91	Lactotransferrin	LTF	[Swiss-Prot: P02788]	*√*	
92	Lamin-A/C	LMNA	[Swiss-Prot: P02545]	*√*	
93	Leucine-rich alpha-2-glycoprotein	LRG1	[Swiss-Prot: P02750]		X
94	Leukocyte elastase inhibitor	SERPINB1	[Swiss-Prot: P30740]	*√*	
95	Long palate, lung and nasal epithelium carcinoma-associated protein 1	LPLUNC1	[Swiss-Prot: Q8TDL5]		X
96	Ly6/PLAUR domain-containing protein 3	LYPD3	[Swiss-Prot: O95274]		X
97	Macrophage-capping protein	CAPG	[Swiss-Prot: P40121]		X
98	Moesin	MSN	[Swiss-Prot: P26038]	*√*	
99	Mucin-16	MUC16	[Swiss-Prot: Q8WXI7]		X
100	Mucin-5 AC	MUC5AC	[Swiss-Prot: P98088]		X
101	Mucin-5B	MUC5B	[Swiss-Prot: Q9HC84]	*√*	
102	Myeloperoxidase	MPO	[Swiss-Prot: P05164]	*√*	
103	Myosin-9	MYH9	[Swiss-Prot: P35579]	*√*	
104	Neuroblast differentiation-associated protein AHNAK	AHNAK	[Swiss-Prot: Q09666]	*√*	
105	Neutrophil gelatinase-associated lipocalin	LCN2	[Swiss-Prot: P80188]	*√*	
106	NSFL1 cofactor p47	NSFL1C	[Swiss-Prot: Q9UNZ2]		X
107	Peptidyl-prolyl cis-trans isomerase B	PPIB	[Swiss-Prot: P23284]		X
108	Periplakin	PPL	[Swiss-Prot: O60437]	*√*	
109	Peroxiredoxin-1	PRDX1	[Swiss-Prot: Q06830]	*√*	
110	Peroxiredoxin-2	PRDX2	[Swiss-Prot: P32119]		X
111	Peroxiredoxin-5, mitochondrial	PRDX5	[Swiss-Prot: P30044]		X
112	Peroxiredoxin-6	PRDX6	[Swiss-Prot: P30041]		X
113	Phosphoglycerate mutase 1	PGAM1	[Swiss-Prot: P18669]	*√*	
114	Plasma protease C1 inhibitor	SERPING1	[Swiss-Prot: P05155]		X
115	Plasminogen	PLG	[Swiss-Prot: P00747]		X
116	Plastin-2	LCP1	[Swiss-Prot: P13796]	*√*	
117	Polymeric immunoglobulin receptor	PIGR	[Swiss-Prot: P01833]	*√*	
118	Profilin-1	PFN1	[Swiss-Prot: P07737]	*√*	
119	Proteasome subunit alpha type-1	PSMA1	[Swiss-Prot: P25786]		
120	Proteasome subunit alpha type-3	PSMA3	[Swiss-Prot: P25788]		
121	Proteasome subunit alpha type-4	PSMA4	[Swiss-Prot: P25789]		X
122	Proteasome subunit alpha type-5	PSMA5	[Swiss-Prot: P28066]		X
123	Proteasome subunit alpha type-6	PSMA6	[Swiss-Prot: P60900]		X
124	Proteasome subunit beta type-1	PSMB1	[Swiss-Prot: P20618]		
125	Proteasome subunit beta type-4	PSMB4	[Swiss-Prot: P28070]		
126	Proteasome subunit beta type-6	PSMB6	[Swiss-Prot: P28072]		
127	Proteasome subunit beta type-8	PSMB8	[Swiss-Prot: P28062]		
128	Protein disulfide-isomerase	P4HB	[Swiss-Prot: P07237]	*√*	
129	Protein disulfide-isomerase A3	PDIA3	[Swiss-Prot: P30101]		X
130	Protein disulfide-isomerase A4	PDIA4	[Swiss-Prot: P13667]		X
131	Protein disulfide-isomerase A6	PDIA6	[Swiss-Prot: Q15084]		X
132	Protein DJ-1	PARK7	[Swiss-Prot: Q99497]		
133	Protein S100-A8	S100A8	[Swiss-Prot: P05109]	*√*	
134	Protein S100-A9	S100A9	[Swiss-Prot: P06702]	*√*	
135	Pyruvate kinase isozymes M1/M2	PKM2	[Swiss-Prot: P14618]	*√*	
136	Ras GTPase-activating-like protein IQGAP1	IQGAP1	[Swiss-Prot: P46940]		X
137	Selenium-binding protein 1	SELENBP1	[Swiss-Prot: Q13228]		
138	Serotransferrin	TF	[Swiss-Prot: P02787]	*√*	
139	Serpin B6	SERPINB6	[Swiss-Prot: P35237]		X
140	Serum albumin	ALB	[Swiss-Prot: P02768]	*√*	
141	Sulfhydryl oxidase 1	QSOX1	[Swiss-Prot: O00391]		X
142	Superoxide dismutase [Cu-Zn]	SOD1	[Swiss-Prot: P00441]	*√*	
143	Synaptic vesicle membrane protein VAT-1	VAT1	[Swiss-Prot: Q99536]		X
144	Thioredoxin	TXN	[Swiss-Prot: P10599]	*√*	
145	Transaldolase	TALDO1	[Swiss-Prot: P37837]	*√*	
146	Transitional endoplasmic reticulum ATPase	VCP	[Swiss-Prot: P55072]		X
147	Transketolase	TKT	[Swiss-Prot: P29401]		X
148	Triosephosphate isomerase	TPI1	[Swiss-Prot: P60174]	*√*	
149	Vimentin	VIM	[Swiss-Prot: P08670]	*√*	
150	Vinculin	VCL	[Swiss-Prot: P18206]	*√*	
151	Vitamin D-binding protein	GC	[Swiss-Prot: P02774]	*√*	
152	Vitronectin	VTN	[Swiss-Prot: P04004]		X
153	Zinc-alpha-2-glycoprotein	AZGP1	[Swiss-Prot: P25311]		X

*Present in the 136 Cervical-Vaginal Fluid Core proteins defined by Zegels et al. [13].

**Proteins that were not identified in the 136 Cervical-Vaginal Fluid Core proteins defined by Zegels et al. [13] but were present in at least one of ten previous studies reviewed by Zegels et al. [10]

### Overlap of “Normal Pap Test Core Proteome” with other CVF proteomic studies

In a comprehensive proteomic analysis of cervical-vaginal fluid (CVF), Zegels et al. [13] determined a set of 136 “CVF Core Proteins” which were present in at least three of the four most comprehensive analyses of the CVF proteome [11,13,15,20]. We compared the lists of proteins that we had identified in the residual Pap test fluid of the 5 individuals (Additional file 2) to the list of “CVF Core Proteins”, and found 132 of the 136 “CVF Core Proteins” were present in at least one of the individual Pap test samples. Furthermore, about half (64) of the 153 proteins listed in our “Normal Pap test Core Proteome” were also present in the “CVF Core Proteome” (Table 2, column 5). An additional 61 of the proteins in our “Normal Pap test Core Proteome” were also found in at least one of ten analyses of CVF proteins enumerated in a recent review [10] (Table 2, last column). These data demonstrate that the use of residual Pap test fluid for the identification of CVF proteins is similar to other sampling and detection methodologies.

### Estimation of protein abundance

We estimated the relative abundance of the proteins identified in the individual and pooled Pap test samples by calculating a normalized spectral abundance factor (NSAF) for each protein (Additional files 1 and 2, last column) that takes into account both the spectral counts for each protein as well as the protein size [13,27,28]. Ten of the “Normal Pap test Core” proteins were among the thirty most abundant proteins in at least five experiments. These proteins include neutrophil gelatinase-associated lipocalin, serotransferrin, lactotransferrin, S100A8 and S100A9, which all play a role in immune response. Albumin, hemoglobin alpha, and hemoglobin beta were also among the ten proteins found in at least five experiments.

## Discussion

This study represents the first publication in which the cell-free residual Pap test fluid has been examined as a source for proteomic profiling of CVF in women with normal cervical cytology. Using pooled samples, we identified more than 700 unique proteins; while in individual Pap test samples more than 300 proteins were identified. By merging proteins identified in the pooled samples with proteins identified in 4 of 5 individual Pap tests analyzed by MS, we determined a “Normal Pap test Core Proteome” of 153 proteins that is similar in composition to that of other proteomic analyses of CVF [10,11,13-15,17,18,20-22].

Previous characterization of the CVF proteome has relied on sampling methods such as Dacron tipped swabs [15,17,19,20], sponges or gauze [11,16], or direct collection of CVF [29,30] or cervical washings [13,14]. Only in the analysis by Zegels [13], who used cervical washings collected during colposcopy, were routine clinical samples utilized for proteomics. In addition, our MS/MS proteomic technique using the individual residual Pap test samples still yielded as many or more protein identifications than previously reported proteomic analyses of CVF, which at most found 685 proteins [10,13]. The use of the FASP protocol for trypsin digestion combined with sensitive instrumentation for the mass spectrometry analysis made the analysis of individual specimens possible.

We used Genome Ontology databases to classify the proteins identified in residual Pap test fluid by cellular localization and biological processes [23,24]. In both the pooled and individual samples, approximately 40% of the proteins identified were localized to the plasma membrane or extracellular compartments. This is similar to other studies of CVF which found approximately 30% of the proteins identified were extracellular or membranous in origin [10,11,13]. Similarly, we also identified many proteins involved in immunity and defense, proteolysis, cell adhesion and numerous cytoskeletal proteins. Among the cytoskeletal proteins, we report several keratin proteins as part of our “Normal Pap test Core Proteome”. While keratins are commonly considered a contaminant in mass spectrometry, cytokeratin intermediate filaments are components of the cornified envelope (CE), a highly crosslinked structure formed beneath the plasma membrane of epithelial cells that serves a barrier function [31]. Additional structural CE proteins, such as involucrin and periplakin, were identified in our study and in other proteomic analyses of CVF [11,13,15,20]. Indeed, Zegels et al. [13] reported that a “large portion” of the proteins identified in their study were CE components, although the identification of cytokeratins was apparently excluded from their analysis. The presence of these and other intracellular proteins in the cell-free residual Pap test fluid is likely due to *in situ* cytolysis, through mechanical disruption, bacterial lysis or autolysis. The cytokeratins identified in the CVF are therefore a reflection of the cellular composition of the female genital tract, which express a distinctive cytokeratin profile [32].

We believe that the majority of cytoplasmic and nuclear proteins that we identified by MS were most likely due to proteolysis that occurred *in situ*, rather than during collection of the clinical sample *per se*. The BD SurePath^TM^ preservative fluid contains ethanol, methanol, isopropanol, and formaldehyde; it was developed to serve as a fixative for cervical cells collected during a liquid-based Pap test. The SurePath^TM^ fixative should diminish (if not eliminate) proteolytic degradation. Fixative solutions may crosslink proteins and nucleic acids, so as to interfere with proteolytic enzymes and potentially inhibit cellular lysis [33,34]. For our purposes of MS-based proteomics, the “fixative” attribute of the SurePath^TM^ preservative fluid proved to be advantageous. Studies have shown that DNA in cervical specimens was stable for human papillomavirus testing when stored in SurePath^TM^ fixative for up to 10 weeks at ambient temperature [35]. The Material Safety Data Sheet for the ThinPrep® PreservCyt Solution states that the cytologic sample can be stored for up to six weeks at 39-99°F [6]. Additional studies have shown that DNA could be extracted and PCR amplified from either SurePath^TM^ or ThinPrep® Pap test samples stored for more than 2.5 years [36]. However, there is a paucity of information to document the stability of proteins in these liquid-based Pap test fixatives. Thus, the formulation of Pap test fixatives that are currently on the market may need to be improved upon to ensure that proteins are not degraded if they are to be analyzed in MS-based proteomic studies.

The relative abundance of proteins in the residual Pap test samples was estimated by NSAF, and revealed that neutrophil gelatinase-associated lipocalin, S100A8 and S100A9 were among the most abundant proteins identified. All three proteins function in innate immunity, a common function of CVF proteins [37,38], and have been previously identified in the CVF proteome [11,13,15,20]. In one study of CVF, a similar NSAF calculation determined that S100A9 was the most abundant CVF protein [13]; however, although S100A9 was identified in every sample we examined, it was among the 30 most abundant proteins in only six of seven samples analyzed.

One potential advantage of using residual Pap test fluid as a source for biomarker discovery is that CVF may not contain the high abundance proteins that impede the identification of low abundance proteins in similar proteomic analyses of serum and plasma. We examined the residual Pap test samples for the presence of serum albumin using Western immunoblot, and found the level of albumin to be substantially lower than in serum. However, when we examined the Pap test samples by mass spectrometry, we identified a large number of peptides specific for albumin in the residual Pap test samples despite having excluded samples with visible blood contamination. In this study, we specifically chose not to deplete the highly abundant proteins from the Pap test samples prior to MS, since our goal was to see whether it would be feasible to perform a limited number of steps of sample manipulation and still identify hundreds of proteins. In addition, when we designed these studies, we were concerned that by depleting the highly abundant proteins, we may also deplete some of the low abundance proteins that bind to albumin or hemoglobin.

While the presence of serum proteins is not directly addressed in other proteomic studies of CVF, serum albumin and several hemoglobin subunits were among the 10 most abundant proteins identified in CVF by Zegels et al. [13], and serum albumin was identified in all ten proteomic studies of CVF compared in the Zegels et al. review [10]. In future studies, the depletion of serum albumin and hemoglobin (as well as other highly abundant serum proteins) may improve the identification of lowly abundant CVF proteins. Furthermore, Pap test samples from women with gynecological conditions may warrant depletion of the highly abundant proteins in order to identify proteins that are differentially expressed.

Importantly, our study demonstrates the feasibility of using residual Pap test samples as a protein source for proteomic analysis of CVF. The ability to use a commonly collected clinical specimen for proteomic studies could pave the way for biomarker discovery for any number of gynecological disorders, as well as the FDA approved use in the screening and detection of cervical cancer, pre-cancerous lesions, atypical cells and other cytologic categories [4]. In addition to cytological examination of cells collected for identification of cervical cancer, Pap test samples are now routinely used to test for the presence of human papilloma virus DNA [39], but could potentially be used for diagnosis of other gynecological diseases.

The long-term goal of the research in our laboratory has been to develop a diagnostic test for the early detection of ovarian cancer. The median age of women who are diagnosed with ovarian cancer is 63 years, with almost 90% of those diagnosed over the age of 45 [40]. In this feasibility study, we chose to use Pap test samples from women who were at least 50 years old, so that we could define the “Normal Pap test Core Proteome” for this population of women who had normal cytology reports. In ongoing studies, we are using Pap test samples from women who are diagnosed with ovarian cancer (all of whom are over 50 years old), with the intent of comparing their proteome to this “Normal Pap test Core Proteome”. For other gynecological conditions, it may be necessary to select a cohort of women with a lower median age to serve as the “normal” healthy control group.

Using an approach similar to ours, two studies examined cervical cytology specimens by MS in order to stratify them according to cervical cancer risk [41] or for the identification of biomarkers of cervical disease [22]. In another study, Kinde et al. [42] reported a technique (termed Safe-SeqS assay) to detect somatic mutations in the DNA of rare tumor cells present in the liquid fixative solution of Pap tests for the identification of gynecological cancers. All three studies used the liquid Pap test sample; however, they examined the cellular component of the Pap test for either DNA mutations that were known to be present in tumors from the same patient [42], or for MS profiles of cytospins [41] or laser capture microdissected cells from ThinPrep slides [22]. In our case, we used sensitive MS methods to examine “cell-free” Pap test fluid to detect proteins that are shed or secreted by cells in the female genital tract, and showed that the Pap test fluid could be used as a source for biomarker discovery. We are very optimistic that state-of-the-art technology for DNA mutations [42] coupled with our MS technology for proteins will one day be used routinely in the clinic for cancer detection, including cervical neoplasms, endometrial endometrioid and serous carcinomas, and serous tubal intraepithelial carcinomas (“STIC”), the putative precursor of ovarian cancers [12]. It will be necessary to more fully explore the sources of biological, technical, and experimental variations in order to define the feasibility of using residual Pap test fixatives for clinical diagnostics.

## Conclusions

We determined that the “cell-free” component of residual Pap test fixative contains a sufficient amount of protein for analysis by MS, and have used it to define the “Normal Pap test Core Proteome”. Since residual Pap test fluid is readily available from millions of patients, it represents a valuable biospecimen source for the identification of protein biomarkers for gynecological diseases and has the potential to change the way that women are routinely tested for gynecological cancers.

## Methods

### Clinical specimens

Clinical specimens were collected per routine procedures using the BD SurePath^TM^ liquid-based Pap test. In the clinic, cervical cells were collected from the ectocervix of healthy women by a physician using a BD broom-like device specifically designed for this purpose. The detachable head of the sampling device was immediately placed into a BD SurePath^TM^ vial, which contains 10 ml of a mixture consisting of 21.7% ethanol, 1.2% methanol, 1.1% isopropanol, and formaldehyde [5]. In the clinical laboratory, the BD SurePath^TM^ vials were shaken to remove cells from the head of the broom-like device, and then 8 ml of the SurePath^TM^ solution underwent automated processing to eliminate debris and distribute a representative portion of cells on a slide in a uniform, even layer. Cells were then stained and examined by a pathologist.

For this study, we obtained deidentified residual (waste) Pap test samples in SurePath^TM^ vials from the University of Minnesota BioNet Tissue Procurement Facility with approval from the IRB (Protocol 1101E94895). At our institution, the SurePath^TM^ vials are stored for one month at room temperature after the Pap test sample has been processed, at which time they were made available for our use in this study. Samples selected for this feasibility study were from women at least 50 years old (median age of 58 years; ranging from 50–76 years) with normal cytology and without visible blood contamination.

### Sample processing

**Figure 4:**
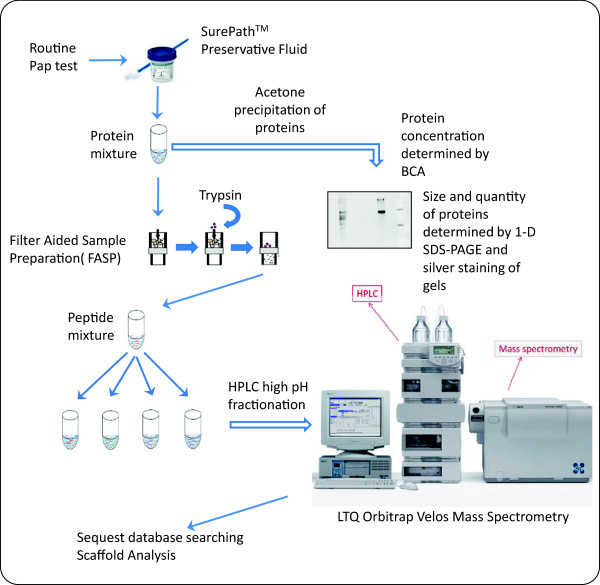
**Diagrammatic representation of the workflow involved in Pap test sample preparation for MS analysis.** Following a routine Pap test, the SurePath^TM^ vials were sent to cytopathology for a diagnosis. Excess residual SurePath^TM^ fluid from women with normal cytology was sent to the research laboratory. Protein concentration was determined by the BCA protein assay, proteins were precipitated with acetone, and visualized with silver stain by SDS-PAGE. Precipitated proteins were also trypsin digested and processed by FASP, and peptides were run on HPLC followed by MS. Data was analyzed by Sequest database searching and Scaffold analysis.

The workflow of Pap test samples from processing to MS/MS analysis is depicted in Figure 4. SurePath^TM^ vials were vortexed to resuspend proteins that may have settled during the one-month of storage at room temperature in the cytology laboratory, as well as to release cells/proteins from the cervical sampling device that remained in each vial. The residual fluid was centrifuged for 5 min at 800 × g to pellet the cells. Protein concentration in the cell-free SurePath^TM^ fluid was determined using the bicinchoninic acid (BCA) protein assay in microplates (Pierce Protein Research Products, Rockford, IL) according to the manufacturer’s instructions.

### Polyacrylamide gel electrophoresis and Western immunoblots

Five μg of protein from the cell-free Pap test fluid of over 100 individuals was concentrated by acetone precipitation. Briefly, proteins were precipitated from the fluid with 5 volumes of ice cold acetone overnight at -20°C, and then centrifuged for 20 min at 6,000 × g at 4°C. The pellet was solubilized in gel loading buffer [0.0625 M Tris, pH 6.8; 1% SDS (w/v); 0.05% bromophenol blue (w/v); 10% glycerol (w/v); 1% β-mercaptoethanol (v/v)]. Proteins were separated on a 4-20% gradient Tris–HCl Criterion^TM^ gel (BioRad, Hercules, CA) in Tris-glycine buffer [0.1% SDS (w/v), 25 mM Tris, 192 mM glycine, pH 8.3]. Gels were either silver stained as previously described [43,44] or electroblotted using a Criterion^TM^ Blotter (BioRad) onto a polyvinylidene difluoride (PVDF) membrane (Pall Corporation, Pensacola, FL) in transfer buffer (12.5% methanol, 25 mM Tris base, 192 mM glycine, pH 8.0). Western immunoblots were blocked with 5% non-fat dried milk in phosphate buffered saline (PBS) with 0.05% Tween-20, and then incubated with an affinity purified polyclonal antibody raised in rabbits against human serum albumin (AB-40AP, Advanced Targeting Systems, San Diego, CA). After washing, blots were incubated with a horseradish peroxidase conjugated secondary antibody (Pierce), and visualized with chemiluminescence using SuperSignal West Femto Maximum Sensitivity substrate (Pierce). Images were collected by exposure to Kodak ×500 film (Midwest Scientific, Valley Park, MO).

### Filter aided sample preparation

Equal volumes of SurePath^TM^ fixative from 40 randomly selected normal Pap test samples were pooled and acetone precipitated as above, yielding ~ 250 ug of protein. Precipitated proteins for pooled and individual samples were resuspended in 10 mM Tris, pH 7.6, 4% sodium dodecyl sulfate (SDS). Pooled and individual samples (~50-100 ug protein) were prepared for mass spectrometry by Filter Aided Sample Preparation (FASP) using Nanosep Omega centrifugal devices with a 10 K MW cut off (Pall Corp., Port Washington, NY) as a reaction vessel [45,46]. Samples were reduced by the addition of 10 mM Tris(2-carboxyethyl)phosphine (TCEP) at room temperature. Proteins were alkylated with 50 mM iodoacetamide (Sigma-Aldrich, St. Louis, MO) and digested with trypsin (enzyme: protein ratio 1:100) overnight at 37°C. Peptides were desalted with C18 stage tips (Thermo Scientific, West Palm Beach, FL) and dried under vacuum.

### High pressure liquid chromatography fractionation

Trypsin digested samples were fractionated offline by high pH reverse phase chromatography [47] using a MAGIC 2002 high pressure liquid chromatography (HPLC) instrument (Michrom BioResources, Inc., Auburn, CA) and C18 Gemini-NX column [150 mm × 2 mm i.d., 5 um particle, 110 Å pore size (Phenomenex, Torrence, CA)]. The flow rate was maintained at 100 μL/min using Buffer A (10 mM ammonium formate pH 10) and Buffer B (10% Buffer A: 90% acetonitrile) at 5-35% gradient for 60 minutes, followed by 35-60% gradient for 5 minutes. Absorbance was monitored at 215 and 280 nm wavelengths. Thirty-two fractions were collected at 2-minute intervals and vacuum-dried. Fractions containing peptides were resuspended in loading solvent (98% water: 2.0% acetonitrile: 0.01% formic acid) prior to analyzing by mass spectrometry.

### Mass spectrometry and database searching

Approximately 1–1.5 ug (5 ul) aliquots of the fractions of pooled or individual Pap test samples were run on a LTQ Orbitrap Velos mass spectrometer (Thermo Fisher Scientific, Inc., Waltham, MA) as described previously [48] with the exception that the higher-energy collisional dissociation (HCD) activation energy was 0.1 ms. Sequest (version 27, rev 12) was used for peptide matching and protein identification. MS/MS data were searched against a human Uniprot database (version_042012) plus common contaminants (thegpm.org/crap/index, 109 proteins), and a concatenated reversed sequence database for a total of 293,452 proteins. The search parameters were Fragment Tolerance: 0.80 Da (monoisotopic), Parent Tolerance: 0.073 Da (monoisotopic), carbamidomethyl as the fixed modification, methionine oxidation as the variable modification, trypsin digestion, two missed cleavages allowed, and 95% confidence for the detected protein threshold.

The dta/out files generated by Bioworks were analyzed in Scaffold (version _3.6.2, Proteome Software Inc., Portland, OR) to validate MS/MS based peptide and protein identifications and for relative protein quantitation. Peptide identifications were accepted if they could be established at >95.0% probability as specified by the Peptide Prophet algorithm [49]. Protein identifications were accepted if they could be established at >99.0% probability by the Protein Prophet algorithm [50], and contained at least 2 identified peptides. Rates of false positive identifications were estimated using the target-decoy method [51]. False positive rates were < 1.0% for all experiments.

### Calculations of the relative abundance of proteins

For semi-quantitative estimation of the abundance of proteins, we determined the total count of MS/MS spectra for each protein. To correct the spectral count for differences in protein size, we normalized by dividing the number of counted spectra to the length of proteins (number of observable peptides) in in-silico trypsin digestion [13,27,28]. We then calculated the Normalized Spectral Abundance Factor (NSAF) as follows:

$$\mathrm{NSA}F_{N}=\left[\frac{S_{k}/L_{k}}{\sum_{i=1}^{N}\left(S_{i}/L_{i}\right)}\right]\times \;1000$$

Where S is the number of spectral counts for protein *k,* L is the length of protein *k* and *N* is the total number of proteins identified. We multiplied by 1000 for convenience in presentation of small numbers.

### Classification of proteins by cellular localization and biological function

The proteins identified by MS were classified by cellular localization and biological function using PANTHER database (version 8.1) [24] and Ingenuity IPA (version 2013, 17199142, Ingenuity® Systems, http://www.ingenuity.com) and the UniProtKB Protein Knowledge database.

## Abbreviations

2D: Two dimensional; BCA: Bicinchoninic acid; CE: Cornified envelope; CVF: Cervical-vaginal fluid; FASP: Filter aided sample preparation; GO: Gene Ontology; HPLC: High pressure liquid chromatography; MALDI: Matrix assisted laser desorption/ionization; MS: Mass spectrometry; NSAF: Normalized spectral abundance factor; PAGE: Polyacrylamide gel electrophoresis; Pap test: Papanicolaou test; PBS: Phosphate buffered saline; PVDF: Polyvinyl difluoride; SDS: Sodium dodecyl sulfate; STIC: Serous tubal intraepithelial carcinomas.

## Competing interests

The authors declare that they have no competing interests.

## Authors’ contributions

KLMB and SA performed sample preparation, developed protocols, participated in MS data analysis, and drafted the manuscript. MAG conceived of the study and participated in the design of the study for access to clinical specimens. KH processed samples, determined protein concentrations, ran samples on SDS-PAGE, and performed Western immunoblotting. TJG conceived of the study and participated in the design of the mass spectrometry experiments. SEP conceived of the study and participated in the design of the study for access to residual clinical specimens. APNS conceived of the study, participated in the design and oversight of the study, and helped to draft and write the manuscript. All authors read, edited, and approved the final manuscript.

## Supplementary Material

Additional file 1 MS/MS data of pooled Pap test samples. Click here for file

Additional file 2 MS/MS data of Pap test fluid from 5 individuals. Click here for file
